# Partial Oral Therapy for Infective Endocarditis Among Adult Infectious Diseases Physicians in the United States: An Emerging Infections Network Survey

**DOI:** 10.1093/ofid/ofaf580

**Published:** 2025-09-15

**Authors:** Jack W McHugh, Larry M Baddour, Supavit Chesdachai, Susan E Beekmann, Philip M Polgreen, Walter R Wilson, Daniel C DeSimone

**Affiliations:** Division of Public Health, Department of Medicine, Infectious Diseases and Occupational Medicine, Mayo Clinic, Rochester, Minnesota, USA; Division of Public Health, Department of Medicine, Infectious Diseases and Occupational Medicine, Mayo Clinic, Rochester, Minnesota, USA; Division of Public Health, Department of Medicine, Infectious Diseases and Occupational Medicine, Mayo Clinic, Rochester, Minnesota, USA; Division of Infectious Diseases, Carver College of Medicine University of Iowa, Iowa City, Iowa, USA; Division of Infectious Diseases, Carver College of Medicine University of Iowa, Iowa City, Iowa, USA; Division of Public Health, Department of Medicine, Infectious Diseases and Occupational Medicine, Mayo Clinic, Rochester, Minnesota, USA; Division of Public Health, Department of Medicine, Infectious Diseases and Occupational Medicine, Mayo Clinic, Rochester, Minnesota, USA

**Keywords:** bacteremia, infective endocarditis, oral therapy, oral antibiotics, partial oral therapy, step-down therapy

## Abstract

**Background:**

Recent clinical trial evidence supports broader use of partial oral therapy (POT) for infective endocarditis (IE), yet real-world uptake in the U.S. has not been investigated.

**Methods:**

Adult infectious diseases (ID) physician members of the Infectious Diseases Society of America Emerging Infections Network were surveyed in April–May 2025. A 10-item instrument captured frequency of POT, organism-specific influence, decision factors, barriers, and facilitators.

**Results:**

Among 1531 members, 516 (34%) responded; 452 (88%) of them managed IE. POT was uncommon: 16% never used, 53% used in ≤10% of cases, and only 10% used in >25% of patients. Frequent POT rose with caseload (23% in physicians treating >50 IE cases year vs ≤9% in lower-volume groups, *P* < .001) and with fewer years in clinical practice (13% in <5 yrs vs 5% in ≥25 yrs, *P* = .013). Comfort with POT depended on the pathogen: 66% were comfortable switching for *Streptococcus spp.*, 52% for Gram-negative bacilli, 19% for methicillin-resistant *Staphylococcus aureus*. Three quarters of those who used POT finished with a single agent. In people who inject drugs, 34% of physicians often or always considered an oral regimen. Availability of an active oral agent (75%) and the pathogen involved (69%) were the leading decision drivers; principal barriers were fear of relapse (72%), adherence concerns (53%), and insufficient evidence (48%). Respondents most desired clearer guidelines (75%) and additional data (71%).

**Conclusions:**

U.S. adult ID physicians adopt POT for IE sparingly. Updated IE treatment guidelines, additional clinical trial data, and broader access to complex outpatient antimicrobial therapy services may facilitate wider adoption.

Infective endocarditis (IE) is characterized by a hospital mortality of ∼20% and one-year mortality exceeding 30% despite advances in diagnostics and antimicrobial therapy [1]. Standard management has historically relied on prolonged intravenous (IV) antimicrobial therapy, typically delivered through outpatient parenteral antimicrobial therapy (OPAT) programs. While OPAT offers significant advantages over prolonged hospitalization [2], IV antibiotics can increase cost, expose patients to catheter-related complications, and demand increased monitoring from healthcare teams [3, 4].

Previous studies suggest that not all patients need to complete therapy intravenously. In 2019, the partial oral treatment of endocarditis (POET) trial demonstrated noninferiority in left-sided cases [5], but it excluded patients with methicillin-resistant *Staphylococcus aureus,* had few immunocompromised patients, patients with Gram-negative IE, and enrolled only five people who inject drugs (PWID). Several recent observational cohorts from Europe and the United States support these findings and suggest that oral therapy may be feasible beyond the narrower population enrolled in POET [6–9].

In response, the 2023 European Society of Cardiology (ESC) guidelines assigns a class IIa recommendation for partial oral therapy (POT) once a minimum of 10 days of IV therapy have been completed (7 days following valve surgery, where relevant), provided strict stability criteria have been satisfied [10]. A 2022 American Heart Association (AHA) Scientific Statement on IE in PWID emphasized its practicality in select patients [11], while noting persistent evidence gaps. In contrast, the AHA IE guidelines, last updated in 2015 [12], acknowledged POT as a potential option for right-sided MSSA IE in PWID, but stopped short of a recommendation, and the extent to which U.S. infectious disease (ID) physicians have adopted POT into their practice remains unknown.

To understand how these factors affect practice, we used the Infectious Diseases Society of America (IDSA) Emerging Infections Network (EIN). EIN is a provider-based sentinel surveillance system supported by the Centers for Disease Control and Prevention. Membership includes ID physicians, pharmacists, advanced practice clinicians, and public-health professionals. The network conducts rapid surveys that bridge clinical practice and public-health priorities and represents roughly one-fifth of practicing United States ID physicians [13] and this platform was used to survey its US adult ID physician members. Our objectives were to quantify current use of POT for IE, identify organism-specific practices, and delineate barriers and facilitators.

## METHODS

We distributed a survey related to POT in IE via the IDSA EIN. Three ID physicians (J.M., L.M.B, and D.C.D.) developed the initial survey questions, which were then refined in collaboration with EIN leadership (S.B. and P.P.), and pilot tested in conjunction with the authors. The survey consisted of 10 questions, and the complete survey instrument is included in the Supplementary Data. The survey was distributed via the EIN in April 2025 with reminders sent at one and two weeks. The survey closed on 13 May 2025. Participation was voluntary and confidential, and the University of Iowa institutional review board deemed the project nonhuman subjects research and thus exempt.

Denominators varied because not all EIN members responded to all questions. For some questions, respondents could select >1 option, resulting in some percentages totaling >100%. Descriptive statistics and statistical significance assessed using Fisher's exact tests were conducted, considering *P* = .05 the threshold of significance. Ninety-five percent confidence intervals (95% CIs) for proportions were generated with the Wilson score method. The described United States regions followed Census Bureau definitions. Figures were created using RStudio 2024.12.1 (RStudio Team, 2024).

## RESULTS

### Respondent Characteristics

Of 1531 eligible EIN members, 516 responded (34% response rate) and 452 (88% of respondents) reported managing IE. This represents ∼4.8% of all U.S. ID physicians, based on 2023 data from the American Medical Association [14], and provides a 95% CI with a margin of error of approximately ±4%. Nearly all (99.2%) respondents lived in the United States, most commonly in the South Atlantic (20%), Atlantic (16%), West North Central (14%), Pacific (14%), and East North Central (13%) regions. Career stage:13% had <5 years of experience, 32% 5–14 years, 24% 15–24 years, and 31% ≥ 25 years since fellowship. Forty-two percent practiced in university hospitals and 47% in community or nonuniversity teaching sites, whereas Veterans Affairs/Department of Defense, city–county, and outpatient-only settings accounted for the remaining 11%. Most physicians (78%) managed ≥6 IE cases annually and 9% handled >50 (Table 1).

**Table 1.** Characteristics of 516 EIN Member respondents

Respondent Characteristic	*N* (%)
Region	
New England	39 (8)
Atlantic	83 (16)
East North Central	68 (13)
West North Central	73 (14)
South Atlantic	101 (20)
East South Central	17 (3)
West South Central	32 (6)
Mountain	28 (5)
Pacific	71 (14)
Canada and Puerto Rico	4 (0.8)
Years’ experience since ID fellowship	
< 5	69 (13)
5–14	166 (32)
15–24	124 (24)
≥ 25	157 (31)
Primary Hospital Type	
Community	125 (24)
Nonuniversity teaching	117 (23)
University	217 (42)
Veterans Affairs of Department of Defense	31 (6)
City/County	24 (5)
Outpatient only	2 (0.4)
Number of IE cases managed annually	
None	64 (12)
1–5	52 (10)
6–20	196 (38)
21–50	157 (31)
> 50	47 (9)

### Frequency and Predictors of Partial Oral Therapy

**Figure 1.**
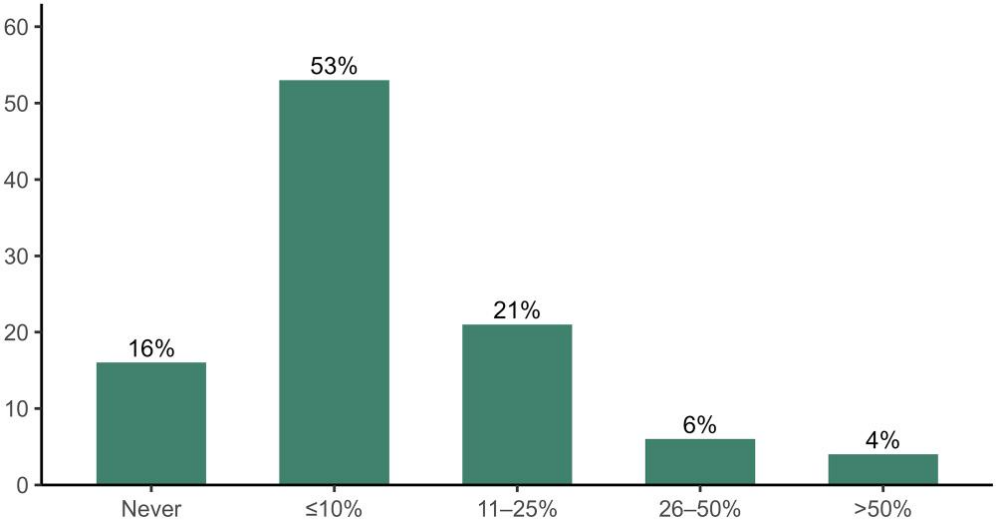
Frequency of partial oral therapy for infective endocarditis management.

Among the 452 ID physicians who manage IE, 16% (95% CI 13%–19%) report never transitioning to oral therapy and 53% (49%–58%) report doing so in ≤10% of cases. Only 10% (8%–13%) switch in more than one quarter of patients: 6% (4%–8%) in one-quarter to one-half of cases and 4% (2%–6%) in more than half (Figure 1).

Table 2 demonstrates that switching frequency was strongly associated with annual IE caseload (Fisher's exact *P* < .001). Frequent or very frequent use of POT was reported by 23% of physicians managing >50 cases per year, compared with 7% for 21–50, 8% for 6–20, and 10% for 1–5. Nonuse was highest among low-volume physicians (31% for 1–5 cases) and lowest among those managing >50 cases (6%). Comfort with POT was also associated with fewer years in clinical practice (*P* = .01): 13% of physicians with <5 years reported frequent or very frequent use, compared with 5% among those with ≥25 years. Nonuse was reported by 9% with <5 years versus 24% with >25 years. Hospital type was not significantly associated with POT frequency (*P* = .06), although frequent or very frequent use was somewhat higher in university and nonuniversity teaching hospitals (10%) than in community (6%) or VA/DoD sites (4%).

**Table 2.** Variation in Partial Oral Therapy Prescribing According to Caseload, Years of Experience, and Practice Site

Practice Variations By Caseload Per Y				
	1–5 (%)	6–20 (%)	21–50 (%)	50+ (%)
Never	16 (31)	37 (19)	15 (10)	3 (6)
Rarely (<10%)	19 (36)	116 (59)	90 (57)	17 (36)
Occasionally (10–25%)	12 (23)	28 (14)	41 (26)	16 (34)
Frequently (25–50%)	7 (6)	8 (4)	8 (5)	7 (15)
Very frequently (>50%)	2 (4)	7 (4)	3 (2)	4 (9)

Practice variations by y of experience				
	<5 y (%)	5–14 y (%)	15–24 y (%)	25+ y (%)
Never	6 (9)	16 (10)	21 (19)	28 (24)
Rarely (<10%)	36 (54)	90 (57)	63 (57)	53 (46)
Occasionally (10–25%)	16 (24)	32 (20)	20 (18)	29 (25)
Frequently (25–50%)	3 (4)	13 (8)	5 (4)	5 (4)
Very frequently (>50%)	6 (9)	7 (5)	2 (2)	1 (1)

Practice variations by practice site					
	City/County (%)	Community (%)	VA/DoD (%)	Nonuniversity teaching (%)	University Teaching (%)
Never	2 (10)	23 (22)	5 (18)	18 (17)	23 (12)
Rarely (<10%)	12 (60)	54 (50)	15 (54)	61 (58)	100 (52)
Occasionally (10–25%)	2 (10)	23 (21)	48 (25)	17 (16)	48 (25)
Frequently (25–50%)	0	3 (3)	1 (3)	9 (8)	13 (7)
Very frequently (>50%)	4 (20)	4 (4)	0	1 (1)	7 (4)

### Comfort, Decision Drives, and Barriers to Oral Therapy

Of 444 respondents, 66% were comfortable with POT for streptococcal IE and 52% for Gram-negative bacillary IE; confidence fell to 30% for HACEK organisms infection, 28% for methicillin-susceptible *S. aureus*, 27% for coagulase-negative staphylococci, and 21% and 19% for enterococcal IE and methicillin-resistant *Staphylococcus aureus* (MRSA) IE, respectively. Among the 306 physicians who use oral therapy, 75% complete treatment with a single high-bioavailability agent unless dual coverage is pathogen-mandated (for example *Coxiella burnetii* or Bartonella IE); 25% routinely prescribe two oral drugs.

**Figure 2.**
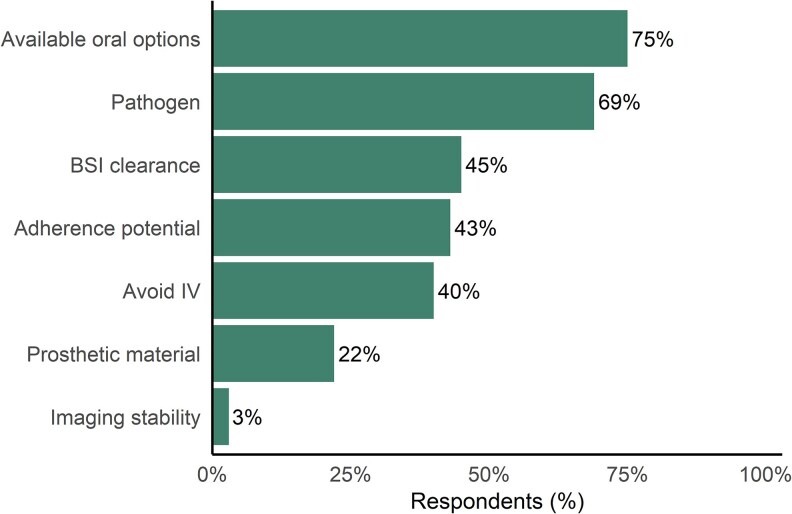
Please select the three factors that most influence your decision whether to partial oral therapy for infective endocarditis. BSI, bloodstream infection; IV, intravenous.

**Figure 3.**
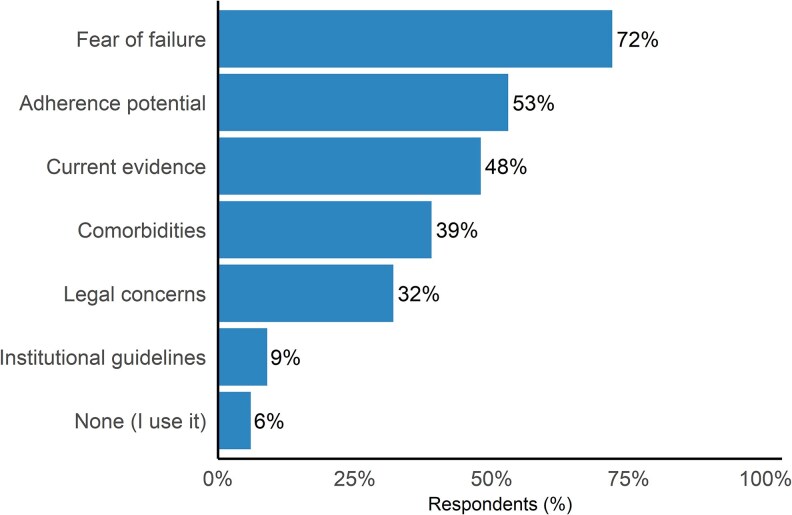
What are the primary issues that prevent you from prescribing partial oral therapy for infective endocarditis?

When asked to rank their three leading decision factors (424 responses and 32 selected >3), 75% chose availability of a suitable oral agent and 69% cited the pathogen (Figure 2). Clinical stability followed, with 45% choosing bloodstream clearance and 43% weighing the patient's ability to adhere. Avoiding prolonged IV access (40%), prosthetic material (22%), and imaging evidence of stability (3%) were less frequently ranked. Eighteen of 30 free-text comments described complex cases, including patient-directed discharges or situations where follow-up with OPAT was unlikely or not feasible. Barriers echoed these priorities: of 447 respondents, 72% cited fear of relapse, 53% adherence concerns, 48% insufficient evidence, and 39% comorbidities that could impair absorption (Figure 3). Medico-legal worry was reported by 32%, institutional restrictions by 9%, and only 6% noted no major barriers. Of 55 free-text responses, 11 expressed openness to prescribing POT but described hesitation due to prevailing institutional norms favoring IV therapy; five specifically called for updated guidelines. Seven cited the lack of data for MRSA, and four raised concerns about use in immunocompromised patients. Eight mentioned challenges with adherence to oral therapy, and six highlighted concerns about toxicities associated with prolonged use of linezolid or trimethoprim–sulfamethoxazole (TMP–SMX).

### Partial Oral Therapy in People Who Inject Drugs

Regarding POT in PWID, of 445 respondents, 8% never and 18% rarely consider an oral switch, 38% occasionally contemplate it, and 26% often or 8% always do so, meaning 74% of physicians are open to oral therapy in PWID. Among 45 free-text responses, 13 described using oral options when IV therapy was not feasible. A recurring theme was “concern about adherence to oral antibiotics,” and when oral regimens were used, the inability to monitor for toxicities in this patient population. To address these challenges, 8 respondents favored long-acting glycopeptides (eg, dalbavancin and oritavancin) as a practical alternative. Three noted that IV therapy can facilitate access to harm reduction services, and one highlighted potential drug interactions between linezolid or rifampin and medications used in addiction treatment such as methadone.

### Guideline Influence and Implementation Needs

When asked specifically about guidelines (*n* = 452), 6% reported no influence, 18% slight, 39% moderate, 26% strong, and 9% very strong influence. In response to what would increase adoption of oral therapy (447 responses; multiple selections allowed), 75% endorsed clearer guidelines or consensus statements, and 71% cited the need for more prospective trial data. Practical supports were also emphasized: 35% wanted improved adherence monitoring, 32% broader access to well-tolerated oral agents or insurance coverage, and 24% institutional protocols. Among 20 free-text comments, five highlighted the need for complex outpatient antimicrobial therapy (COpAT) given the monitoring requirements for commonly used oral agents. Six again emphasized the lack of data for MRSA. Two noted cost concerns, including the need for prior authorization for oral linezolid.

A free text option soliciting general comments (*n* = 80) revealed several recurring themes. First, 14 respondents raised concerns for the toxicities of oral medications and a lack of dedicated COpAT resources to monitor patients on oral therapy, with some citing a desire for COpAT teams to ensure adherence, and others citing the need for more-readily available therapeutic drug monitoring (TDM) for these agents as was done in the POET trial. Fourteen respondents cited the need for additional data in specific populations, including 7 for MRSA and two for immunocompromised patients. Twelve respondents emphasized the need for guidelines to help shift entrenched institutional practices and, in some cases, address medicolegal concerns. Ten commented on the emergence of lipoglycopeptides as an alternative to daily IV therapy and their preference for these agents over oral therapy.

## DISCUSSION

Our survey of U.S. adult ID physicians demonstrates that POT for IE is used sparingly. Among physicians who manage IE, over two-thirds reported never or rarely making an oral switch, and only 10% reported doing so in more than one quarter of cases. Comfort with POT was more common in streptococcal and Gram-negative IE, with steep declines for other pathogens including MRSA and enterococci. Use was more common among physicians with larger IE caseloads and fewer years in practice, suggesting that clinical volume and recent training may facilitate adoption. Despite these trends, adoption remained low across all subgroups, underscoring the physicians’ persistent caution surrounding this practice.

Our findings both confirm and extend those of the recent international survey by Mathé et al [15], which included 74 clinicians, 78% based in Europe and only 1 respondent in North America. In their study, 50% never used POT and just 5% applied it in >50% of cases. Comfort likewise centered on streptococci (50%) and fell to 19% for *Staphylococcus aureus*, mirroring the pattern seen in our cohort. Both surveys identified the same leading deterrents: perception of limited evidence, outdated or absent guidelines, and fear of relapse.

Considering recent data from both observational studies and the POET trial, why does POT uptake remain low amongst U.S. physicians? While the absence of U.S.-based guidelines and prevailing institutional culture are important drivers, our survey highlights that fear of treatment failure, often related to limited pathogen-specific data, adherence and medicolegal concerns, remains the leading deterrent. This central concern is reflected in several specific themes raised by respondents.

First, many physicians cited evidence gaps, particularly the exclusion of immunocompromised patients, those with MRSA, and the limited enrollment of PWID in the POET trial [5]. High-quality data for MRSA is lacking, and ongoing randomized IE trials (RODEO-I and RODEO-II) exclude MRSA [16], and the SNAP trial does not include a POT arm for IE [17]. In the two largest observational studies to date, Freling et al (*n* = 34 for POT in MRSA) and Wildenthal et al (*n* = 32 for POT in MRSA) reported similar outcomes for POT and completion IV therapy for MRSA, although these cohorts included other organisms and results were not stratified by organism [6, 9]. While our survey suggests that some physicians are willing to extrapolate from other pathogens when a highly bioavailable agent is used and stability criteria are met, the need for randomized, MRSA-specific data remains clear.

With respect to PWID, Wildenthal et al (2023) specifically focused on outcomes in complicated *Staphylococcus aureus* bloodstream infection (BSI). They found after ≥ 10 days of IV therapy, POT with high-bioavailability agents was noninferior to completion IV therapy, whereas discharge without oral cover was associated with higher failure rates; noting that of the 32 cases of complicated MRSA BSI reported, the number of IE cases was not specified. The 2022 AHA consensus statement for PWID acknowledges the growing evidence for POT in this population, although stops short of recommending routine POT [11]. Our survey highlights mixed practices in this cohort, with many favoring the use of long-acting glycopeptides where available, citing concern for adherence to oral agents, although data for these agents is primarily from small observational studies [18].

A second major barrier was concern about adherence, toxicity, and the laboratory burden associated with high-bioavailability oral agents (linezolid, TMP–SMX, fluoroquinolones, and rifampin). Respondents repeatedly called for COpAT frameworks to manage these risks. Most centers now have mature OPAT programs, but COpAT—defined as oral courses >30 days or those needing scheduled lab surveillance, remains early in its adoption. When implemented, such programs can match OPAT's cost and bed-day savings while providing the monitoring our respondents deem essential [19], and adequately resourced teams may also be empowered to cater to the needs of PWID. Several respondents also advocated for more routine availability of TDM for oral agents. TDM for linezolid and TMP–SMX (sulfamethoxazole component), in particular, may mitigate toxicity and facilitate extended treatment durations [20, 21].

This study has limitations. First, participation was voluntary; EIN members represent ∼1 in 5 U.S. ID physicians, and our 34% response rate introduces selection and nonresponse bias. University-affiliated physicians were over-represented; such centers manage more complex IE cases yet have greater COpAT capacity, so referral bias could either inflate or depress reported use of POT. All responses were self-reported and therefore susceptible to recall and social-desirability bias. The survey treated all Gram-negative bacilli as a single category, obscuring distinctions for *Pseudomonas aeruginosa* and other resistant pathogens. We did not capture the timing of the IV-to-oral switch, information that may influence both safety and clinician comfort. Other key stakeholders; cardiologists, cardiac surgeons, pharmacists, and pediatric ID specialists were not surveyed, limiting generalizability. Finally, we collected no patient-level follow-up data, preventing assessment of relapse or treatment failure after POT and leaving uncertain whether reported practice patterns translate into favorable outcomes.

In conclusion, POT for IE is deployed sparingly in the sample of adult ID physicians in the U.S. Uptake is highest among early career physicians and high-volume centers but remains modest overall. Broader adoption will hinge on stronger evidence, clearer society and institutional guidance, and reliable outpatient monitoring infrastructure. Future trials should enroll challenging populations, including patients with MRSA and enterococcal infections, PWID, and immunocompromised patients, with prespecified IV lead-in criteria and standardized switch triggers. Randomized designs, or rigorously matched comparisons to continued IV therapy with 90-day and 1-year outcomes are warranted. Updated, interdisciplinary guidelines endorsed by the IDSA that specify patient selection, outline monitoring protocols, and identify research priorities are needed to advance safe, wider use.

## Supplementary Material

ofaf580_Supplementary_Data

## Supplementary Data

Supplementary materials are available at *Open Forum Infectious Diseases* online. Consisting of data provided by the authors to benefit the reader, the posted materials are not copyedited and are the sole responsibility of the authors, so questions or comments should be addressed to the corresponding author.
