# Supplementary material for: Partial Oral Therapy for Infective Endocarditis Among Adult Infectious Diseases Physicians in the United States: An Emerging Infections Network Survey
Source: Open Forum Infect Dis. 2025 Sep 15;12(9):ofaf580. doi: 10.1093/ofid/ofaf580 (PMC12459244; doi:10.1093/ofid/ofaf580)
Supplement: ofaf580_Supplementary_Data [file ofaf580_supplementary_data.docx]

**Survey Cover Letter**

**Current Management Approaches for Infective Endocarditis**

Infective endocarditis (IE) has historically been managed with prolonged courses of parenteral antibiotics, reflecting long-standing clinical practices and guideline recommendations ([AHA guidelines](https://www.idsociety.org/practice-guideline/endocarditis-management/) were last updated in 2015). Recently, emerging evidence has highlighted the feasibility, safety, and efficacy of transitioning patients with IE to oral antibiotics once a clinical response has been achieved to complete the treatment course ([NEJM 2019](https://www.nejm.org/doi/full/10.1056/NEJMoa1808312)). The degree to which infectious disease clinicians have adopted transitional oral therapy into routine clinical practice is currently unknown. Understanding current prescribing practices will influence future research and guideline development.

**We aim to better understand current prescribing practices and perceptions among infectious disease clinicians regarding the use of transitional oral antibiotic therapy in IE patients.**

**
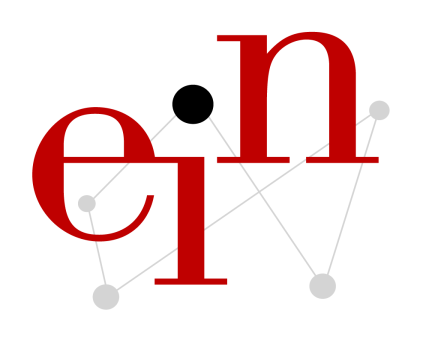
**

**Infectious Diseases Society of America**

**Emerging Infections Network Survey**

Three emailed requests to answer this query were sent to adult ID EIN physician members (4/24, 4/30 and 5/7/2025).

**Question 1. How many cases of infective endocarditis (IE) do you manage annually?**

None

1-5

6-20

21-50

>50

**Question 2. How often do you transition from intravenous to oral antibiotic therapy for patients with IE?**

Never

Rarely (≤10% of cases)

Occasionally (11-25% of cases)

Frequently (26-50% of cases)

Very frequently (>50% of cases)

**Question 3. Please select the THREE factors below that most influence your decision whether to transition to oral therapy for IE:**

Clearance of bloodstream infection

Pathogen

Availability of oral antibiotic options with appropriate spectrum

Adherence potential to oral therapy with follow-up

Imaging evidence of stable or improving valvular infection

Prosthetic valve or prosthetic valve material

Desire to avoid prolonged intravascular use

Open-text field provided for additional comments

**Question 4. For which pathogens do you feel comfortable transitioning to oral therapy?**

N/A, do not transition to oral therapy

MSSA

MRSA

Streptococcus spp.

Enterococcus spp.

Coagulase-negative staphylococci

HACEK (*Haemophilus*, *Aggregatibacter*, *Cardiobacterium*, *Eikenella*, *Kingella*)

Gram-negative bacilli

**Question 5. The POET trial used transitional oral antibiotic therapy with two antibiotics (combination therapy). In your practice, which best describes your usual approach?**

I do not use transitional oral antibiotic therapy

I always use two oral antibiotics until completion of therapy

I typically use a single oral antibiotic unless the pathogen specifically

requires two antibiotics (e.g. *C. burnetii*, *Bartonella* IE)

**Question 6. What are the primary issues that prevent you from prescribing transitional oral therapy for IE?**

Lack of sufficient evidence

Concerns about patient adherence

Institutional guidelines or restrictions

Fear of treatment failure or recurrence

Patient comorbidities (e.g. gastrointestinal absorption issues)

Medicolegal issues

None, I use transitional oral therapy regularly

Open-text field provided for additional comments

**Question 7. Which choice best describes your approach to transitioning from IV to oral therapy in people who inject drugs (PWID) with IE?**

I never consider an oral switch

I rarely consider an oral switch

I occasionally consider an oral switch

I often consider an oral switch

I always consider an oral switch

Open-text field provided for additional comments

**Question 8. How strongly do current guidelines (e.g. AHA, ESC) influence your decision to consider transitional oral therapy for infective endocarditis?**

Not at all

Slightly

Moderately

Strongly

Very strongly

**Question 9. What would increase the likelihood of incorporating transitional oral therapy into your practice?**

N/A, will not use transitional oral therapy

Clearer guidelines or consensus statements

More prospective clinical trial data

Institutional protocols or support

Access to oral antibiotic regimens with appropriate coverage

Improved methods to monitor patient adherence

Open-text field provided for additional comments

**Question 10. Do you have any additional comments or insights regarding the use of transitional oral antibiotic therapy for IE?**
